# Dinophysis Toxins: Distribution, Fate in Shellfish and Impacts

**DOI:** 10.3390/toxins11070413

**Published:** 2019-07-16

**Authors:** Beatriz Reguera, Juan Blanco

**Affiliations:** 1 Instituto Español de Oceanografía (IEO), Centro Oceanográfico de Vigo, Subida a Radio Faro 50, 36390 Vigo, Spain; 2 Centro de Investigacións Mariñas, Xunta de Galicia, Pedras de Corón S/N, 36620 Vilanova de Arousa, Spain

Several planktonic dinoflagellate species of the genus *Dinophysis* produce one or two groups of lipophilic toxins: (i) okadaic acid (OA) and its derivatives, the dinophysistoxins (DTXs), and (ii) pectenotoxins (PTXs) [1,2,3]. The OA and DTXs, known as diarrhetic shellfish poisoning (DSP) toxins, are acid polyethers that inhibit the protein phosphatase and have diarrheogenic effects in mammals [4,5]. The PTXs are polyether lactones, some of which are hepatotoxic to mice by intraperitoneal injection [6]. The toxicity of pectenotoxins has been questioned since they are not toxic when ingested orally [7]. Filter feeding bivalves retain toxic planktonic microalgae and other suspended matter, acting as vectors of the toxins through the food web. Bivalves contaminated with DTXs are a threat to public health. Shellfish resources exposed to DTXs and other toxic syndromes need to be monitored for early detection of the toxins and their causative agents and subjected to regulations aimed to protect public health.

Forty years after the identification of *Dinophysis fortii* as the causative agent of severe gastrointestinal outbreaks in Japan [1], toxins produced by a few species of *Dinophysis* have been recognized, in terms of persistence and distribution, as the main threat to intensive shellfish exploitation in western Europe, eastern Japan, and to a lesser extent in southern Chile and New Zealand. Recently, *Dinophysis* events have emerged in traditionally “DSP-toxin free” areas (e.g., eastern and north-western USA, the Pacific coast of Mexico, South China Sea). Increased monitoring and regulation may explain certain cases, but some models include *Dinophysis* as a potential winner in global warming scenarios [8], although without taking into account species-specific requirements [9].

The monitoring of *Dinophysis* species and their toxins in shellfish started in the early 1980’s. The old standard mouse bioassay detected and quantified, as okadaic acid equivalent (OA eq.) units, a “cocktail” of lipophilic toxins, and needed 24 to 48 h observation of the experimental animal. The high performance liquid chromatography (HPLC) method developed by the group of Yasumoto [10] and its adaptation to analyse picked cells of *Dinophysis* [11] revealed that species of this genus produced two groups of toxins with different chemical structures and toxic effects: (i) okadaates (OA and dinophysistoxins) and (ii) pectenotoxins—only the former have diarrhetic effects, while the latter are not even regulated in some countries. Other lipophilic toxins, such as yessotoxins and azaspiracids, and even non-toxic fatty acids causing false positives, were co-extracted with *Dinophysis* toxins, leading to complex matrices for the analyses. The next breakthrough was the development of liquid chromatography coupled to mass spectrometry (LC-MS). During his plenary talk at the 8th International Conference on Harmful Algae, Vigo, 1997, Mike Quilliam forecasted this new analytical tool would replace all the other methods [12]. Two years earlier he had shown how the extraction procedure of the time led to toxin profiles of hydrolized precursors of the OA and DTXs [13]. In the same period, Maestrini [14] identified the main gaps in knowledge concerning the biology and population dynamics of *Dinophysis* species. These gaps included questions about the life cycle, nutrition (including the inability to grow *Dinophysis* in laboratory cultures), and the physical-biological interactions explaining their patchy populations. 

Twenty year after these advances, considerable progress has been gained through: (i) the use of sampling strategies which follow the cell cycle and dynamics of low-density and patchy field populations of *Dinophysis* spp. [15,16]; (ii) the application of single-cell manipulations coupled to new molecular and analytical techniques, and finally (iii) the successful establishment of mixotrophic cultures of *Dinophysis* fed the ciliate *Mesodinium rubrum* [17]. Still the main problems faced to monitor *Dinophysis* spp and their toxins are: (i) taxonomic uncertainties with traditional methods, to identify species which are morphologically similar but with different toxic potential; (ii) large differences in toxin profiles and cellular content found between strains of the same species, even in the same location; (iii) to improve predictive capabilities of the occurrence of *Dinophysis* species and their toxins in shellfish; (iv) to develop cost-efficient monitoring systems for the control of shellfish toxins in different molluscs with their specific metabolic responses. Despite these uncertainties, a “*Dinophysis* trigger level” based on cell densities is still widely used in monitoring systems. Different toxins from *Dinophysis* cells/fragments, their grazers, and detritus derived from faecal pellets are ingested by shellfish, affecting their absorption, transformation and elimination in a species-specific manner, and a large proportion are released into the water [18,19,20]. All these processes, which play key roles in the impact of toxic outbreaks on shellfish resources, are poorly known, in particular from a metabolic and genomic point of view. Further, the direct effects of *Dinophysis* toxins on the growth and survival of shellfish species feeding on them have received little attention.

This special issue contains original contributions that advance our knowledge of the distribution and impact of *Dinophysis* toxins on the shellfish industry worldwide. A wide range of topics are covered, from monitoring and regulation of DSP toxins to *Dinophysis* population dynamics, laboratory cultures and the kinetics of uptake, transformation and impact of the toxins in shellfish. Four papers present long (>20 years) times series of monitoring data from regions in Europe and Oceania suffering blooms of *D. acuminata*/*D. acuta* every year. The impact of DSP events in Ireland, Scotland and Spain, with strains with toxin profiles dominated by OA and DTXs, contrasts with their lower impact in New Zealand, with strains with profiles dominated by PTXs. Results from these countries confirm the need for a shellfish species-specific strategy to control the impact of DSP outbreaks, and a site-specific analysis of the response of *Dinophysis*-related outbreaks to climate variability. A paper with the first report of *Dinophysis* toxins in Perú, from LC-MS analyses of individually isolated cells, shows that classification problems persist within the “*D. acuminata* complex”. This problem is also pointed out in the paper from southwest Spain. Two articles deal with the population dynamics, autoecology and the concept of niche segregation for co-occurring toxic species in Reloncaví fjord, southern Chile and the Galician Rías, northwest Spain, and a paper from Brazil describes interactions between *Dinophysis* and its ciliate prey, as well as toxin transfer through the food web during an exceptional bloom of the “*D. acuminata* complex”. Interactions with the prey *Mesodinium rubrum*, its effects on growth and toxin production in mixotrophic laboratory cultures, and considerations/suggestions to optimize mass cultures of *Dinophysis* are dealt with in three contributions.

Contributions from Japan, the pioneer country with the longest records of detection of *Dinophysis* toxins, include a review of the toxin profiles of different *Dinophysis* species with current analytical tools, as well as statistical considerations on DSP toxin monitoring and their anatomical distribution in shellfish. The effects of DSP toxins on shellfish are explored with advanced molecular techniques, RNA sequencing analysis and transcriptomics. Finally, different aspects of the kinetics of DSP toxin accumulation and depuration in shellfish, including predictive models, are investigated in a full review and in contributions about metabolic changes in shellfish and the effect of suspended particulate matter in toxin accumulation.
